# Explanatory Modeling of Tuberculosis Treatment Outcomes: The Role of Community Engagement and Clinical Governance

**DOI:** 10.3390/ijerph23040511

**Published:** 2026-04-16

**Authors:** Ntandazo Dlatu, Lindiwe Modest Faye

**Affiliations:** 1 Walter Sisulu Institute for Clinical Governance, Healthcare Administration, School of Public Health, Faculty of Medicine and Health Sciences, Walter Sisulu University, Mthatha 5099, South Africa; 2 TB and Associated Research and Innovation Platform (TARIP), WSU-TB Research Group, School of Laboratory Medicine and Pathology, Faculty of Medicine and Health Sciences, Walter Sisulu University, Mthatha 5099, South Africa; lfaye@wsu.ac.za

**Keywords:** tuberculosis, drug-resistant TB, treatment outcomes, community engagement, clinical governance, predictive modeling, logistic regression, decision tree analysis, programmatic factors, resistance phenotype, pre-XDR/XDR-TB, rural health systems, South Africa

## Abstract

**Highlights:**

**Public health relevance—How does this work relate to a public health issue?**

- Drug-resistant tuberculosis (DR-TB) remains a critical public health challenge in rural South Africa, where treatment outcomes are influenced by both biological resistance and health system factors.
- This study examines how community engagement and clinical governance interact with clinical predictors to shape treatment outcomes using real-world program data.

**Public health significance—Why is this work of significance to public health?**

- The findings demonstrate that severe resistance phenotypes and programmatic conditions significantly affect DR-TB treatment success, highlighting the need for integrated clinical and system-level approaches.
- The study advances explanatory predictive modeling as a tool for understanding how structural health system processes influence outcomes beyond individual patient-level factors.

**Public health implications—What are the key implications or messages for practitioners, policymakers, and/or researchers in public health?**

- Strengthening clinical governance and community engagement strategies can improve treatment adherence, care coordination, and overall program performance in DR-TB settings.
- Enhancing routine health information systems to capture governance and community-level indicators is essential for improving program evaluation and predictive modeling in public health.

**Abstract:**

**Background:** Treatment adherence and outcomes for drug-resistant tuberculosis (DR-TB) continue to be subpar in rural South Africa, where structural health system limitations, comorbid conditions, and diverse resistance patterns make clinical management more challenging. This study aimed to assess how demographic, clinical, and programmatic factors, including a Community Engagement–Clinical Governance (CE–CG) implementation period, affect DR-TB treatment outcomes using explanatory predictive modeling. **Methods:** A retrospective cohort study was conducted using routine program data from 694 DR-TB patients. A complete-case analysis was performed for multivariable modeling (*n* = 282). Logistic regression and decision tree models were used to examine the relationships between treatment success and selected predictors, including age, sex, treatment regimen, resistance phenotype, comorbidities, and the CE–CG implementation period. Model discrimination and performance were evaluated using receiver operating characteristic (ROC) curves, pseudo-R^2^ statistics, likelihood ratio tests, and multicollinearity diagnostics. **Results:** The cohort had a mean age of 40.7 years, and 58.8% of patients were male. Overall treatment success was 59.9%. Severe resistance phenotypes were rare (1.7%) but clinically significant. Comparative analysis showed no notable demographic or outcome differences between included and excluded patients, indicating minimal selection bias. In adjusted models, treatment initiation during the CE–CG implementation period was significantly linked to lower odds of treatment success (adjusted odds ratio [aOR] = 0.443; 95% CI: 0.240–0.818; *p* = 0.009). Severe resistance phenotypes were strongly negatively associated with treatment success (aOR = 0.303; *p* = 0.056). Logistic regression models had limited discriminatory ability (AUC: 0.523–0.548), while the decision tree model showed modest improvement (AUC: 0.626). Overall, the model’s explanatory power was limited (pseudo-R^2^ = 0.029), although no evidence of multicollinearity was found. **Conclusions:** Programmatic implementation periods and resistance severity were important factors associated with treatment outcomes in this rural DR-TB cohort. Although model discrimination was modest and explanatory power was limited, the findings provide useful insights into structural and programmatic vulnerabilities that affect treatment success in real-world settings. Strengthening clinical governance, improving routine program documentation, and incorporating more granular adherence, social, and governance indicators into routine data systems may improve both program evaluation and future predictive modeling.

## 1. Introduction

Drug-resistant tuberculosis (DR-TB) remains a major global public health challenge, disproportionately affecting low- and middle-income countries [1,2,3]. In high HIV-prevalence settings, particularly in rural regions of sub-Saharan Africa, treatment outcomes are shaped by a complex interplay of biological resistance, patient-level vulnerabilities, and health system capacity. Despite advances in molecular diagnostics and improved treatment regimens, unfavorable outcomes, including treatment failure, loss to follow-up, and death, remain common among patients with DR-TB [4,5,6,7]. While pathogen-related factors, such as resistance phenotype, are well-established determinants of prognosis, structural and programmatic elements are increasingly recognized as critical in shaping treatment trajectories [8,9,10,11]. In resource-limited rural settings, challenges related to adherence support, continuity of care, patient tracing, and integration of TB–HIV services remain persistent. These contextual challenges underscore the importance of clinical governance and community engagement as key structural determinants of treatment success [12]. Clinical governance frameworks emphasize accountability, quality improvement, monitoring, and systematic oversight of healthcare delivery, whereas community engagement focuses on building trust, strengthening adherence, and enabling context-responsive care. Health systems are increasingly adopting integrated community engagement–clinical governance (CE–CG) approaches to enhance tuberculosis program performance [13,14,15,16]. The conceptualization of community engagement and clinical governance (CE–CG) in this study is informed by established health systems and implementation science frameworks. Within the World Health Organization health systems framework, CE–CG can be understood as contributing to core system functions, including service delivery, health workforce performance, information systems, and leadership and governance. In this context, community engagement supports responsiveness, trust, and continuity of care, while clinical governance strengthens accountability, quality assurance, and oversight within healthcare systems [12]. In addition, the CE–CG approach aligns with the Donabedian model, which conceptualizes healthcare quality in terms of structures, processes, and outcomes. Clinical governance can be viewed as strengthening structural and organizational components (e.g., monitoring systems, supervision, and accountability mechanisms), whereas community engagement primarily operates at the process level (e.g., adherence support, patient tracing, and community-based care). Together, these elements influence treatment outcomes by shaping how care is delivered and experienced within real-world program settings. From an implementation science perspective, CE–CG reflects a complex intervention operating within a dynamic health system, where interactions between context, mechanisms, and implementation processes shape outcomes. Governance frameworks that emphasize accountability, stewardship, and system responsiveness further support this perspective, highlighting the roles of institutional arrangements and community relationships in shaping program effectiveness. These approaches may include community health worker-led tracing, structured adherence counseling, community-based follow-up, routine program monitoring, and coordinated TB–HIV care pathways. However, evaluating the effects of governance and community engagement interventions remains methodologically challenging. These mechanisms often operate through complex, multidimensional, and relational processes that are not easily captured using traditional causal models, particularly when relying on routinely collected program data [17,18,19,20]. In this context, predictive modeling techniques can be applied within an explanatory analytical framework to explore how demographic, clinical, and programmatic factors interact within real-world health systems. While most existing modeling studies in DR-TB have focused on individual-level prognostic tools, fewer have examined how structural and program-level factors may be associated with treatment outcomes. Predictive performance in tuberculosis outcome modeling varies considerably across studies, depending on the quality and completeness of available data, the modeling approach used, and the context in which models are developed. Studies using routinely collected clinical and programmatic data have often reported only modest discrimination, particularly where important behavioral, laboratory, and socio-structural predictors are absent. More advanced machine learning approaches, including tree-based ensemble methods, have in some settings demonstrated improved predictive performance compared with traditional regression-based models, especially when richer datasets are available. However, these gains are not always substantial or consistent, and they may come at the cost of reduced interpretability and greater sensitivity to missingness, overfitting, and variable quality. In many real-world program settings where data are incomplete or operationally constrained, the performance gap between regression-based and machine learning approaches may therefore be limited. In such contexts, simpler and more interpretable models may provide comparable practical value while offering greater transparency for health system and program analysis [21,22,23,24]. Predictive modeling offers an opportunity to examine how demographic, clinical, and programmatic factors interact to influence treatment outcomes. In this study, logistic regression and decision tree models were selected to provide complementary analytical perspectives within an explanatory framework. Logistic regression offers a transparent and interpretable approach for estimating adjusted associations between predictors and outcomes, while decision tree models allow exploration of non-linear relationships and potential interaction structures. More advanced machine learning approaches, such as random forests, gradient boosting, and neural networks, have demonstrated improved predictive performance in some settings. However, these methods typically require larger datasets and more comprehensive variable capture and may reduce interpretability, particularly in studies based on routine program data. Given the relatively modest sample size, the extent of missing data, and the study’s focus on explanatory rather than predictive optimization, simpler, more interpretable modeling approaches were considered more appropriate [21]. This perspective aligns with implementation science and systems-oriented analytical frameworks, which emphasize understanding how complex interventions operate within real-world environments where multiple interacting factors influence outcomes. Rather than isolating single causal effects, these approaches prioritize pathways, context, and system-level interactions that shape program performance. Accordingly, this study aimed to apply an explanatory predictive modeling approach to examine how demographic, clinical, and programmatic factors, including community engagement and clinical governance, interact within routine program data to influence treatment outcomes in drug-resistant tuberculosis (DR-TB). Specifically, multivariable logistic regression and tree-based models were used to explore associations between predictors and treatment success. By integrating epidemiological modeling with a clinical governance perspective, this study seeks to generate system-level insight into how structural program environments may shape DR-TB outcomes in rural, high-burden settings.

## 2. Methods

### 2.1. Study Design and Setting

This study used a retrospective cohort design with routinely collected program data from a drug-resistant tuberculosis (DR-TB) treatment program in a rural, high-burden setting in the Eastern Cape Province, South Africa. The analysis was carried out within an explanatory modeling framework to examine treatment outcomes in relation to changes in program structures over time. Specifically, the study examined the implementation of community engagement and clinical governance (CE–CG) strategies as a time-sensitive contextual program exposure, reflecting periods when these strategies were active within the program. CE–CG was not viewed as a separate intervention in a pre–post or quasi-experimental way, but rather as an indicator of the broader service delivery environment in which patients received care. This distinction is important for understanding the findings as associational rather than causal.

### 2.2. Study Population and Data Source

The dataset comprised 694 patient records of individuals diagnosed with drug-resistant tuberculosis (DR-TB) and registered for treatment at public sector tuberculosis treatment facilities serving predominantly rural and high-burden communities in the Eastern Cape Province, South Africa, between January 2018 and March 2020. This 27-month observation period encompassed routine program conditions during which service delivery structures evolved, including the phased operationalization of the community engagement and clinical governance (CE–CG) strategies. The study, therefore, captured patients managed under differing program contexts over time, consistent with its interest in temporal variation in program implementation. Data were extracted from the routine tuberculosis program database. They included demographic variables (age and gender), clinical characteristics (drug-resistance phenotype, treatment regimen at initiation, and comorbidity status), treatment initiation date, and final treatment outcomes. The study population included patients across all age groups, including pediatric cases, as recorded in the routine program database. Treatment outcomes were categorized as favorable (cured or treatment completed) or unfavorable (lost to follow-up, treatment failure, death, transfer out, or still on treatment at the time of analysis), in accordance with standard program reporting definitions. For modeling purposes, the drug-resistance phenotype was classified into severe resistance (pre-extensively drug-resistant tuberculosis [pre-XDR-TB] or extensively drug-resistant tuberculosis [XDR-TB]) and other resistance patterns. The latter category included rifampicin-resistant tuberculosis (RR-TB), isoniazid-resistant tuberculosis (INH-R), multidrug-resistant tuberculosis (MDR-TB) without additional second-line resistance, and other non-severe resistance profiles as defined by routine program diagnostic classification.

### 2.3. Data Handling and Variable Selection

Several clinically relevant variables, including HIV status, baseline smear or culture results, adherence indicators, and socio-economic characteristics, were excluded from the multivariable analysis due to incomplete or inconsistent recording in the routine program dataset. Inclusion of these variables would have substantially reduced the analytic sample and may have introduced additional uncertainty through imputation under poorly understood missing-data mechanisms. Given the relatively modest sample size and the extent of missingness across several routinely collected variables, a complete-case analysis approach was adopted for multivariable modeling. Although this approach reduced the effective sample size and may have introduced bias if data were not missing completely at random, it was considered the most transparent and methodologically conservative strategy within the constraints of the available routine program data. Variable selection for the final multivariable model was guided by biological plausibility, data completeness, and relevance to routine program implementation, ensuring that included predictors were both epidemiologically meaningful and reliably captured within the health system context. The community engagement and clinical governance (CE–CG) variable was included as a program-level contextual exposure intended to capture temporal variation in the implementation environment. Specifically, CE–CG was operationalized as a time-based indicator reflecting whether patients were managed during periods when CE–CG strategies were active within the program. This variable does not represent a specific intervention assignment or a formal pre–post comparison but rather serves as a proxy for broader organizational and service-delivery conditions over time. It therefore does not directly measure governance structures or discrete community engagement activities. Consequently, associations involving CE–CG should be interpreted as contextual and observational, rather than causal. Alternative approaches, including multiple imputation, were considered to address missing data. However, the observed missing-data patterns were heterogeneous and, in some instances, likely reflected systematic differences in data recording practices rather than random omission. In the absence of strong evidence supporting a missing-at-random assumption, the use of multiple imputation would require unverifiable assumptions regarding the missing-data mechanism and model specification. Under these conditions, complete-case analysis was considered a more appropriate and methodologically defensible approach, despite its known limitations. The implications of this decision, including potential bias and reduced statistical power, are explicitly addressed in the study’s interpretation.

### 2.4. Operationalization of Community Engagement and Clinical Governance (CE–CG)

Community engagement and clinical governance (CE–CG) were operationalized as a program-level contextual variable reflecting the intended incorporation of governance- and community-oriented support processes into routine drug-resistant tuberculosis (DR-TB) care. This operationalization was intended to capture variation in the broader organizational and service-delivery context in which patients received care, rather than to represent individual-level intervention exposure or a temporal before-and-after comparison.

In this study, CE–CG referred to program elements conceptually aligned with improving the quality, coordination, accountability, continuity, and oversight of care, including patient tracing, adherence counseling, community-based follow-up, TB–HIV service coordination, and routine program oversight. However, these processes were not directly measured at the individual patient level, nor was their implementation fidelity, intensity, or effectiveness systematically assessed in the routine dataset. Accordingly, the CE–CG variable should be interpreted as reflecting the program’s intended service-delivery orientation rather than confirmed or effective implementation of specific governance or community engagement activities. The CE–CG variable, therefore, served as a contextual explanatory variable rather than a direct measure of intervention delivery or uptake. Associations involving this variable are interpreted as observational and contextual and may remain subject to ecological fallacy, residual confounding, and implementation heterogeneity.

### 2.5. Data Cleaning and Complete-Case Analysis

Continuous variables were assessed for plausibility and converted to numeric format where necessary. Age was centered before regression modeling to improve interpretability and reduce the risk of multicollinearity. For multivariable modeling, a complete-case analysis was performed, excluding records with missing values for any of the modeling variables (age, gender, treatment regimen, treatment outcome, CE–CG implementation period, resistance phenotype, or comorbidity status). This approach was selected because several key variables exhibited substantial missingness, and the underlying missing-data mechanism could not be confidently assumed to be missing-at-random (MAR). Although multiple imputation was considered, it was not implemented because the combination of a relatively modest sample size, inconsistent recording patterns, and uncertainty regarding the missing-data process could have introduced additional bias and instability into the model estimates. While complete-case analysis preserves internal consistency of the analytic dataset, it may reduce statistical power and introduce bias if missingness is not completely at random. These limitations were considered in the interpretation of the findings. Candidate variables for modeling were selected a priori based on biological plausibility, clinical relevance, data completeness, and relevance to routine program implementation. Variable inclusion was therefore guided primarily by substantive and epidemiological considerations rather than automated or purely data-driven selection procedures. Variables considered potential confounders were retained in multivariable analyses where conceptually justified and sufficiently complete within the analytic dataset. The modeling strategy was designed to examine associations between routinely recorded patient- and program-level factors and treatment outcomes, while also exploring the predictive utility of the available variables. The structure informed model specification of the available data and the need to balance interpretability with predictive performance. Predictors were entered as defined in the routine dataset or according to clinically meaningful grouped categories established before analysis.

Given the relatively small complete-case sample, model development was undertaken cautiously to reduce the risk of overfitting. A single train–test split was used to assess out-of-sample predictive performance; however, this approach may yield unstable performance estimates in smaller samples. Cross-validation was not implemented in the present analysis and should be considered in future work to improve the robustness of model evaluation. Accordingly, predictive performance metrics should be interpreted as exploratory rather than definitive indicators of model generalizability.

### 2.6. Descriptive Analysis

Descriptive statistics were calculated for the entire dataset before applying complete-case exclusion criteria to give an overview of the study population. Continuous variables were summarized using means and standard deviations (SD), while categorical variables were reported as frequencies and percentages. To evaluate potential selection bias from complete-case analysis, comparisons were made between included and excluded patients. Independent-samples *t*-tests were used for continuous variables (age), and chi-square tests were used for categorical variables (gender and treatment outcome). In addition to testing for statistical significance, effect sizes were calculated to measure the strength of differences, using Cohen’s d for continuous variables and Cramér’s V for categorical variables. This combined approach allowed for the assessment of both statistical and practical significance. Logistic regression and a decision tree model were chosen to offer complementary analytical insights: a parametric, interpretable framework for estimating adjusted associations, and a nonparametric approach for exploring potential interaction patterns and nonlinear relationships. The modeling strategy focused on interpretability and alignment with an explanatory analytical framework rather than predictive accuracy. More advanced techniques, such as penalized regression or ensemble methods, were considered but not used due to the relatively small sample size, missing data, and the risk of overfitting.

### 2.7. Multivariable Logistic Regression

A multivariable logistic regression model was fitted using the complete-case analytic sample to examine associations between demographic, clinical, and program-level factors and treatment success. Predictor variables were selected a priori based on biological plausibility, clinical relevance, program relevance, and data completeness. They were entered simultaneously into the model to estimate their independent associations with the outcome. The model included centered age, gender, the Community Engagement and Clinical Governance (CE–CG) implementation period indicator, treatment regimen at initiation, severe resistance phenotype, and comorbidity status. The treatment regimen at initiation was categorized as short or long, corresponding to the standardized drug-resistant tuberculosis (DR-TB) treatment regimens used within the national tuberculosis program and aligned with World Health Organization (WHO) recommendations. Severe resistance phenotype was defined as pre-extensively drug-resistant tuberculosis (pre-XDR-TB) or extensively drug-resistant tuberculosis (XDR-TB). Adjusted odds ratios (aORs) with corresponding 95% confidence intervals (CIs) were estimated to quantify the independent association of each predictor with treatment success while accounting for the other variables in the model. Variables considered likely confounders on conceptual grounds were retained in the model irrespective of statistical significance, provided data completeness was adequate. Statistical significance was assessed using two-sided *p*-values, with a predefined threshold of 0.05. The regression analysis was undertaken within an explanatory analytical framework rather than as a predictive model for individual prognosis. Emphasis was therefore placed on interpretability, confounder adjustment, and understanding structural relationships within routinely collected program data.

### 2.8. Statistical Modeling and Model Evaluation

Logistic regression and decision tree models were fitted to examine associations between routinely recorded predictors and unfavorable treatment outcomes, and to explore their utility for outcome classification within the complete-case analytic sample. Logistic regression was used as an interpretable baseline model, while the decision tree was included to explore potential nonlinear relationships and interaction structures not explicitly specified in regression-based modeling. The decision tree analysis was intended to complement the explanatory regression model by identifying interpretable interaction patterns, rather than to develop a clinically deployable prediction algorithm. For the logistic regression model, standard diagnostic procedures were undertaken to assess model adequacy, including evaluation of multicollinearity, influential observations, and overall goodness-of-fit. Model performance was assessed primarily in terms of discrimination, with additional consideration given to calibration, defined as the agreement between predicted probabilities and observed outcomes. Given the relatively small complete-case sample and the exploratory nature of the analysis, formal calibration assessment was limited and is therefore interpreted cautiously. For the decision tree model, complexity was restricted to reduce overfitting and improve interpretability. Tree growth was controlled using predefined stopping criteria, including constraints on maximum tree depth, minimum observations required for node splitting, and minimum observations in terminal nodes. No formal post hoc pruning procedure was applied; instead, model complexity was controlled through these pre-specified depth and node-size constraints. The decision tree was therefore treated as an interpretable, exploratory classification tool rather than a fully optimized predictive model. Model evaluation was conducted using a single train–test split to estimate out-of-sample predictive performance. Predictive performance was summarized using the area under the receiver operating characteristic curve (AUC) and classification-based metrics derived from the test set. However, this approach may yield unstable performance estimates on smaller datasets and is less robust than repeated resampling or cross-validation. Accordingly, performance metrics for both models are interpreted as exploratory rather than definitive indicators of generalizable predictive performance, and future analyses should incorporate cross-validation or external validation where feasible.

### 2.9. Comparative Predictive Modeling

To explore whether non-parametric modeling approaches improved discriminative performance, a decision tree classifier was fitted using the same predictor variables as the logistic regression model. Both models were evaluated using a 70/30 train–test split to assess internal model performance. Discriminative ability was quantified using receiver operating characteristic (ROC) curve analysis and the corresponding area under the curve (AUC). Consistent with the study’s explanatory predictive framework, model performance was interpreted descriptively to evaluate internal coherence and structural insight rather than to develop a deployable prognostic prediction tool.

### 2.10. Model Diagnostics and Goodness-of-Fit

Logistic regression model diagnostics included assessing McFadden’s pseudo-R^2^, likelihood-ratio (LR) testing comparing the full model with the intercept-only model and evaluating multicollinearity using the Variance Inflation Factor (VIF).

McFadden’s pseudo-R^2^ was interpreted within the context of routinely collected clinical program data, where modest explanatory power is commonly observed. VIF values below 5 were considered indicative of acceptable levels of multicollinearity among predictors.

### 2.11. Statistical Software

All statistical analyses were conducted in Python version 3.11 within a Jupyter Notebook version 7 environment to ensure reproducibility. Data management and preprocessing were performed using panda’s version 2.1 and NumPy version 1.26. Inferential statistical analyses were conducted with SciPy 1.11 and statsmodels 0.14, while predictive modeling procedures, including decision tree analysis, were implemented with scikit-learn 1.8.0. Figures, including ROC curves and diagnostic plots, were generated using matplotlib version 3.8. Statistical significance was defined a priori as a two-sided *p*-value < 0.05.

## 3. Results

### 3.1. Study Population and Data Completeness

A total of 694 patient records were identified in the tuberculosis program dataset, of these there were 409 (58.9%) male and 285 (41.1%) females patient records. After applying complete-case criteria for all modeling variables (age, gender, regimen at initiation, treatment outcome, CE–CG period indicator, resistance phenotype, and comorbidity status), 282 patients (40.6%) were retained for multivariable analysis. In comparison, 412 patients (59.4%) were excluded due to missing data in one or more variables. Missingness was primarily driven by incomplete documentation of resistance phenotype and comorbidity status, whereas core demographic variables such as age and gender were largely complete. The complete-case dataset was subsequently used for all multivariable modeling analyses.

### 3.2. Baseline Characteristics

The mean age of the cohort (*n* = 694) was 40.69 years (SD = 17.38), with ages ranging from 5 to 78 years, indicating the inclusion of both pediatric and adult populations. The study population was predominantly male (58.9%), while females accounted for 41.1% of the cohort. Although pediatric cases were included, their number was relatively small compared to that of adult patients; therefore, subgroup analysis by age category was not performed. Overall treatment success, defined as cure or treatment completion, was achieved in 59.9% of patients. Most participants (88.8%) were initiated on a short drug-resistant tuberculosis (DR-TB) treatment regimen, while 6.8% received a long DR-TB regimen. Severe resistance phenotypes, including pre-extensive drug-resistant tuberculosis (pre-XDR-TB) and extensively drug-resistant tuberculosis (XDR-TB), were relatively rare, occurring in 1.7% of patients, although they remain clinically significant. The remaining cases comprised non-severe DR-TB categories, including rifampicin-resistant tuberculosis (RR-TB), isoniazid-resistant tuberculosis (INH-R), and multidrug-resistant tuberculosis (MDR-TB) without additional second-line resistance, consistent with routine program classification. A detailed summary of baseline characteristics is presented in Table 1.

### 3.3. Comparison of Included and Excluded Patients

To assess potential selection bias introduced by the complete-case analysis, patients included in the modeling dataset (*n* = 282) were compared with those excluded due to missing data (*n* = 412). No statistically significant differences were observed between the two groups in terms of mean age (*t* = −1.04, *p* = 0.299), gender distribution (χ^2^ = 1.962, *p* = 0.161), or treatment success proportions (χ^2^ = 3.269, *p* = 0.071). Effect size estimates indicated negligible differences between groups. Cohen’s *d* for age was −0.081, while Cramér’s V values were 0.054 for gender and 0.069 for treatment success, suggesting minimal practical imbalance between included and excluded patients. Although 59.4% of records were excluded due to incomplete modeling variables, these findings indicate that the analytic sample remained broadly comparable to the full cohort across key demographic characteristics and treatment outcomes. The observed missingness appeared primarily related to incomplete documentation of specific clinical variables rather than systematic differences in core demographic or outcome measures. However, the possibility of residual bias due to unmeasured clinical or programmatic factors cannot be entirely ruled out. Despite excluding 59.4% of records due to missing data, comparisons between included and excluded patients showed no statistically or practically significant differences in age, gender, or treatment outcomes, suggesting limited evidence of selection bias in the analytic sample.

### 3.4. Multivariable Explanatory Predictive Modeling

A multivariable logistic regression analysis was performed using a complete-case dataset (*n* = 282) to examine the associations between demographic, clinical, and programmatic factors and treatment success. The predictor variables included centered age, gender, treatment regimen at initiation, severe resistance phenotype (pre-XDR/XDR), comorbidity status, and an indicator for the Community Engagement–Clinical Governance (CE–CG) implementation period. The likelihood-ratio test for the full model was not statistically significant, indicating that the model did not significantly improve fit compared to the null model. This suggests limited overall explanatory power of the included predictors within the dataset. Therefore, the results should be viewed as exploratory rather than confirmatory. After adjusting for covariates, treatment initiation during the CE–CG implementation period (≥2024) was significantly associated with lower odds of treatment success (adjusted odds ratio [aOR] = 0.443, 95% confidence interval [CI]: 0.240–0.818, *p* = 0.009). The presence of severe resistance phenotypes (pre-XDR/XDR) was associated with lower odds of treatment success and approached statistical significance (adjusted odds ratio [aOR] = 0.303, 95% confidence interval [CI]: 0.089–1.029, *p* = 0.056). The presence of severe resistance phenotypes (pre-XDR/XDR) was also negatively associated with treatment success and approached statistical significance (aOR = 0.303, 95% CI: 0.089–1.029, *p* = 0.056). In contrast, other covariates, including age, gender, treatment regimen at initiation, and comorbidity status, were not significantly associated with treatment outcomes in the adjusted model (Table 2).

The constant represents the model’s baseline reference level. Age was centered (age_c) by subtracting the sample mean to enhance interpretability and minimize potential multicollinearity.

### 3.5. Comparative Model Performance: Logistic Regression vs. Tree-Based Models

**Figure 1:**
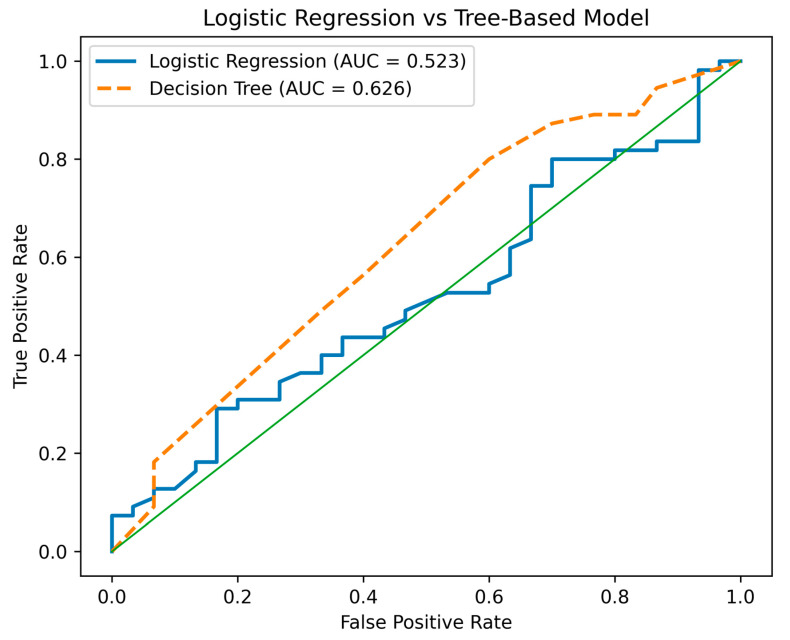
Comparative Model Performance: Logistic Regression vs. Tree-Based Models. Note: The green line is added for clarity.

To evaluate whether non-parametric modeling approaches improved predictive discrimination, a decision tree classifier was developed alongside the logistic regression model, both using the same set of predictor variables. Both models were evaluated using a 70/30 train–test split to assess internal predictive performance. The modest discriminative performance observed (AUC 0.52–0.63) indicates limited predictive utility for individual-level prognosis but provides insight into the structural relationships among clinical and programmatic variables within the dataset. The logistic regression model demonstrated limited discriminative ability, with an area under the receiver operating characteristic curve (AUC) ranging from 0.523 to 0.548, indicating performance only marginally better than chance. In contrast, the decision tree classifier showed modestly improved discrimination (AUC = 0.626), suggesting greater capacity to capture nonlinear relationships and hierarchical interactions among predictors. Despite this relative improvement, overall predictive performance remained modest across both modeling approaches, indicating that the available variables have limited explanatory capacity for predicting treatment success in the current dataset (Figure 1).

**Figure 2:**
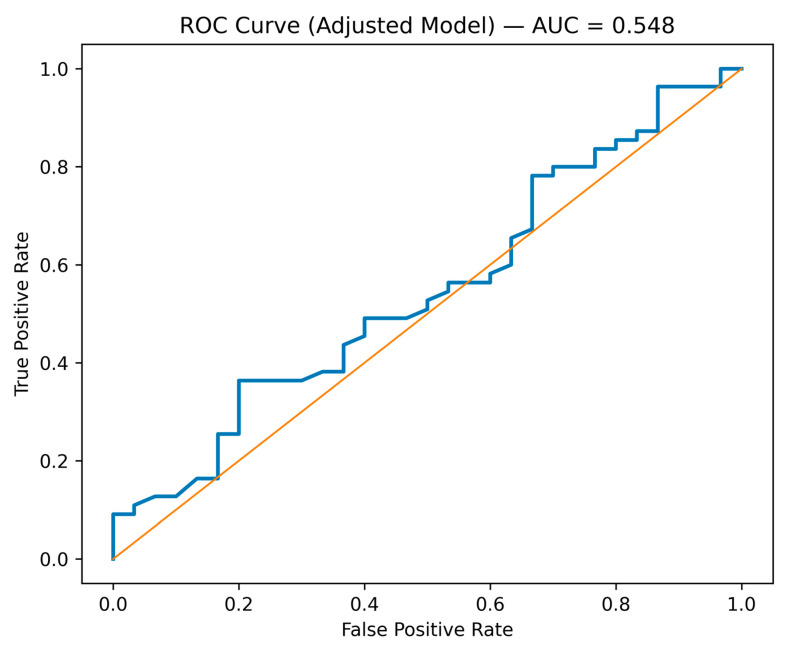
Receiver Operating Characteristic Curve for the Adjusted Explanatory Predictive Model of Treatment Success. Note: The blue line represents the ROC curve of the fitted predictive model, illustrating its ability to discriminate between treatment success and non-success across different threshold values. The orange diagonal line represents the line of no discrimination (area under the curve = 0.5), indicating that the model has no predictive ability (i.e., random classification). The closer the blue curve approaches the upper left corner of the plot, the better the model’s overall predictive performance.

To evaluate whether nonparametric modeling improved discriminative performance, a decision tree classifier was fitted alongside the multivariable logistic regression model, both using the same predictor variables. Both models were assessed using a 70/30 train–test split and receiver operating characteristic (ROC) curve analysis. The logistic regression model demonstrated limited discrimination (AUC = 0.523–0.548), indicating modest ability to distinguish between favorable and unfavorable treatment outcomes. The decision tree model showed improved discrimination (AUC = 0.626), suggesting that hierarchical partitioning may better capture nonlinear relationships and interaction structures within the dataset. Nevertheless, overall performance remained moderate across both approaches. These findings reflect the complementary strengths of modeling strategies. Logistic regression provides interpretable effect estimates (adjusted odds ratios with confidence intervals), supporting inferential clarity and governance-relevant interpretation. In contrast, tree-based models identify structural decision pathways and potential threshold effects without imposing linear assumptions. However, neither approach achieved sufficient discrimination to support individual-level prognostic application. The modest AUC values likely reflect limitations of the available predictor set, including the absence of longitudinal adherence markers, socioeconomic instability measures, and quantified governance-process indicators. Consistent with the study’s explanatory-predictive framework, model performance is interpreted descriptively to assess internal coherence and structural insight rather than to develop externally generalizable prediction tools, as demonstrated in Figure 2.

### 3.6. Model Diagnostics and Goodness-of-Fit

The multivariable logistic regression model, fitted on the complete-case dataset (*n* = 282), included centered age, gender, CE–CG implementation period, regimen at treatment initiation, severe resistance phenotype, and comorbidity status as predictors of treatment success. Model diagnostics showed modest explanatory power with acceptable internal consistency. McFadden’s pseudo-R^2^ was 0.029, indicating a limited explanatory contribution of the predictors compared to the intercept-only model, which is common when analyzing routinely collected clinical program data. Likelihood-ratio testing comparing the full model with the intercept-only model gave χ^2^(6) = 10.70, *p* = 0.098, showing a small, non-significant improvement in model fit. Assessment of multicollinearity revealed Variance Inflation Factor (VIF) values near 1.0 for all predictors, suggesting no problematic collinearity among the independent variables. Overall, these diagnostics support the model’s structural validity while confirming its limited explanatory power. In line with the study’s explanatory modeling framework, the regression model is primarily used as a descriptive tool for examining programmatic and structural factors affecting treatment outcomes rather than as a predictor for individual clinical decisions. A summary of model diagnostics is shown in Table 3.

## 4. Discussion

This study applied an explanatory predictive modeling approach to examine how demographic, clinical, and programmatic factors, including a Community Engagement–Clinical Governance (CE–CG) implementation period, were associated with treatment outcomes in a rural drug-resistant tuberculosis (DR-TB) program. The relatively low model performance (AUC 0.52–0.63; pseudo-R^2^ = 0.029), together with the non-significant likelihood-ratio test, indicates that the available predictors explained only a limited proportion of the observed variation in treatment outcomes. These findings suggest that important determinants of DR-TB outcomes, including adherence behavior, HIV-related factors, socioeconomic vulnerability, and more direct measures of governance and community support, were not adequately captured in the routine dataset. Accordingly, the findings should be interpreted as exploratory and explanatory rather than predictive or causal. Nevertheless, the analysis remains useful in highlighting structural and programmatic patterns that may influence DR-TB care in real-world rural settings. The modeling strategy was intentionally designed within an explanatory analytical framework, prioritizing interpretability and system-level insight rather than predictive optimization. In resource-limited settings, such approaches are increasingly recognized for their value in examining how interactions between clinical factors, program processes, and governance structures influence outcomes [22,24]. This systems-oriented perspective aligns with contemporary approaches in implementation science and health systems research, which emphasize the role of context, mechanisms, and system interactions in shaping program performance [21,23]. As a result, the limited model performance may reflect incomplete measurement of relevant system-level variables rather than the absence of meaningful underlying relationships. Three key findings emerged from the analysis. First, although a substantial proportion of records was excluded due to missing data, the analytic sample remained broadly demographically comparable to the full cohort, suggesting no strong evidence of major selection bias based on the measured variables available for comparison. However, the extent of missing data likely reduced statistical power, may have affected model stability, and does not exclude the possibility of bias arising from unmeasured differences or systematic missingness. Accordingly, the complete-case analytic sample may not fully represent the broader program population. Second, severe resistance phenotypes appeared to be associated with lower treatment success, although this relationship did not reach conventional levels of statistical significance and should therefore be interpreted cautiously. The estimate is best understood as suggestive rather than conclusive. Nonetheless, the observed trend is consistent with existing evidence highlighting the clinical importance of advanced resistance profiles in shaping DR-TB treatment outcomes [25,26]. The modest discriminative performance observed in both logistic regression and decision tree models likely reflects the limitations of routine program data in capturing complex, process-driven interventions. Governance and community engagement mechanisms often operate through relational and organizational pathways such as adherence counseling, community health worker engagement, patient tracing, and integrated service coordination, which are rarely quantified at the individual patient level [27,28,29,30,31,32,33,34,35,36].

Third, the CE–CG implementation period was independently associated with treatment outcomes after adjustment for key covariates; however, this finding requires particularly careful interpretation. The observed association between the CE–CG implementation period and lower treatment success should not be interpreted as evidence of a negative governance effect. Several alternative explanations and sources of bias should be considered. The implementation period likely coincided with broader program transitions, including expansion of diagnostic capacity, increased detection of more complex or advanced DR-TB cases, modifications to treatment regimens, decentralization of care, and evolving case-mix patterns. These concurrent changes may have independently influenced treatment outcomes, introducing temporal confounding and program transition bias. In addition, the CE–CG variable serves as a proxy for program-level change rather than a direct measure of governance processes or intervention fidelity. This introduces the potential for ecological fallacy and residual confounding, as the variable reflects system-level dynamics rather than individual-level exposure. Furthermore, the use of routinely collected program data introduces measurement limitations, including incomplete capture of adherence, comorbidities, and social determinants of health, which may also have confounded the observed association. For these reasons, the CE–CG finding should be interpreted as a contextual and observational signal rather than as a causal estimate of governance effectiveness. These findings are broadly consistent with previous DR-TB modeling studies, which have shown that treatment outcomes are strongly influenced by biological resistance, adherence, and health system factors. At the same time, predictive performance is often constrained by the limited availability of behavioral, social, and program-level variables in routine health datasets. From a program evaluation perspective, the findings also align with implementation science frameworks that conceptualize interventions in terms of context, mechanisms, and outcomes. Within this perspective, the CE–CG implementation period can be understood as a marker of system-level change, reflecting shifts in program context and delivery conditions rather than a discrete intervention exposure. Importantly, governance constructs were not directly measured in this study but were represented through a program-level proxy. This limits the ability to infer specific governance mechanisms and their causal effects, underscoring the need for improved measurement of governance-related processes, including supervision, accountability structures, and community engagement activities. Within this explanatory framework, the findings highlight structural relationships in DR-TB treatment pathways rather than serving as clinically deployable predictive tools. The analysis confirms the dominant influence of biological resistance while also illustrating the limitations of routine program datasets in capturing governance quality and process-level care mechanisms [37,38,39,40,41,42]. Additionally, the absence of key clinical and contextual predictors such as HIV status, microbiological indicators, adherence behavior, and socio-economic factors likely contributed to the model’s limited explanatory power. These variables are well-established determinants of TB treatment outcomes, and their omission reflects data limitations rather than a lack of relevance.

Future research should prioritize integrating more granular and measurable indicators of community engagement and clinical governance, including adherence counseling frequency, community health worker contact rates, clinical review intervals, and TB–HIV service integration metrics. In addition, alternative analytical approaches—such as longitudinal designs, multilevel modeling, penalized regression, ensemble learning, and mixed-methods frameworks—may offer further insight into the complexity of program implementation and patient pathways in DR-TB care, provided that sufficiently complete and high-quality data are available. While the findings offer useful public health insight for strengthening DR-TB programs, their policy implications should be interpreted cautiously. The results highlight the need for improved routine data systems and more robust analytical approaches to better disentangle the effects of concurrent system-level changes, rather than providing definitive evidence of governance effectiveness.

### Strengths and Limitations

This study has several important strengths. First, it applies an explanatory predictive modeling framework to examine treatment outcomes within the broader context of community engagement and clinical governance (CE–CG), extending beyond conventional patient-level prognostic modeling. By integrating epidemiological modeling with a governance-sensitive perspective, the study contributes to a growing body of literature examining how health system structures and processes influence clinical outcomes. Second, the use of routinely collected program data from a real-world DR-TB treatment setting enhances the practical relevance and applicability of the findings, particularly for program evaluation and implementation research in rural, high-burden contexts. Third, the use of complementary modeling approaches, including logistic regression and decision tree analysis, enabled comparisons between parametric and nonparametric methods and provided additional insight into the dataset’s structural relationships. However, several limitations should be considered when interpreting the findings. A substantial proportion of records was excluded due to missing data, resulting in approximately 59.4% of cases being omitted from the complete-case multivariable analysis. Although comparisons between included and excluded patients showed no major differences in observed variables such as age, gender, or treatment outcomes, the possibility of residual selection bias due to unmeasured confounders cannot be ruled out. This reduction in sample size likely decreased statistical power and may have affected the stability and generalizability of the model estimates. The use of complete-case analysis, while methodologically transparent, assumes that missingness is not systematically related to outcomes or key predictors. This assumption cannot be fully verified in this dataset. Although multiple imputations were considered, heterogeneous missing-data patterns and inconsistent recording practices limited confidence in the assumptions required for valid imputation. Accordingly, findings should be interpreted as exploratory and may not fully represent the broader program population. The relatively modest model performance (AUC 0.52–0.63; pseudo-R^2^ = 0.029), together with the non-significant likelihood-ratio test, indicates limited explanatory power of the models. This likely reflects the absence of key determinants of treatment outcomes—such as adherence behavior, HIV status, microbiological indicators, and socio-economic factors—within the routine dataset. These variables are well-established contributors to DR-TB outcomes, and their omission reflects data limitations rather than a lack of relevance. As such, the models should be interpreted primarily as explanatory tools for identifying structural patterns rather than as clinically deployable prediction models for individual-level risk stratification. The operationalization of CE–CG as a program-level time-period indicator represents an additional limitation. While this approach captures contextual changes in the implementation environment, it does not directly measure intervention intensity, fidelity, or individual-level exposure. As a result, the analysis is subject to potential ecological fallacy and residual confounding, and the observed associations may reflect broader system-level transitions rather than direct effects of governance or community engagement interventions. Furthermore, the CE–CG implementation period may have coincided with concurrent programmatic changes, including expansion of diagnostic capacity, decentralization of care, changes in treatment regimens, and evolving patient case mix, which may have independently influenced treatment outcomes and introduced temporal confounding.

The study also did not employ more advanced modeling approaches, such as penalized regression or ensemble learning methods, which may have captured more complex relationships and improved predictive performance. However, given the modest sample size and extent of missing data, the modeling strategy prioritized interpretability, transparency, and stability within an explanatory framework. In addition, the inclusion of pediatric cases introduces clinical heterogeneity, as treatment approaches differ between children and adults; however, the relatively small number of pediatric cases limited the feasibility of age-stratified analyses. Finally, governance constructions were not directly measured. Still, they were represented using a program-level proxy (CE–CG implementation period), which may oversimplify complex governance dynamics and limit the ability to assess specific mechanisms. Future research should incorporate more direct and measurable indicators of governance and community engagement, including supervision intensity, adherence counseling frequency, community health worker contact, and program monitoring processes, to better capture the multidimensional nature of these constructs. Despite these limitations, the study offers important implications for strengthening DR-TB programs in high-burden rural settings. The observed association between severe resistance phenotypes and treatment outcomes reinforces the importance of early detection, accurate resistance profiling, and appropriate regimen selection. Strengthening laboratory capacity and expanding access to rapid molecular diagnostics remain critical priorities. The findings also highlight the importance of reinforcing clinical governance mechanisms, particularly during periods of program transition. Structured approaches, including routine monitoring systems, clinical audits, and multidisciplinary case reviews, may improve coordination, accountability, and responsiveness within TB programs. In addition, the modest model performance observed in this study underscores the need to strengthen routine health information systems. Incorporating more granular indicators of adherence, community engagement, and governance processes, such as adherence counseling frequency, community health worker contact rates, clinical review intervals, and TB–HIV service integration, could enhance both explanatory insight and analytical capacity in future research. The findings further support the continued development of community-engaged TB care models, particularly in rural and resource-limited settings where social vulnerability and access barriers remain significant. Strengthening community-based support systems, including patient tracing, integrated care, and local health worker involvement, may improve treatment outcomes and make TB programs more resilient.

## 5. Conclusions

This study applied an explanatory modeling approach to examine how demographic, clinical, and programmatic factors were associated with treatment outcomes in a rural drug-resistant tuberculosis (DR-TB) program in a high-burden setting. The findings reinforce the importance of biological resistance severity while also illustrating how structural and programmatic conditions may shape treatment outcomes within routine health system environments. The explanatory modeling framework used in this study provides a governance-sensitive analytical perspective for understanding program dynamics rather than a tool for individual-level risk prediction. Although predictive performance was modest, the analysis offers useful insight into how clinical, structural, and programmatic factors may interact in real-world DR-TB care, while also highlighting important limitations in current routine data systems. The observed associations, including those related to the community engagement and clinical governance (CE–CG) implementation period, should be interpreted cautiously. These findings likely reflect complex, overlapping program dynamics, including temporal changes, evolving case mix, and broader system-level transitions, rather than the direct causal effects of CE–CG interventions. Similarly, the observed association between severe resistance phenotypes and poorer outcomes, although consistent with existing evidence, should be interpreted cautiously given the limited explanatory power of the models and the constraints of the available data. Important limitations, including substantial missing data, the use of proxy measures for governance, and the absence of key clinical, behavioral, and socio-contextual variables, restrict the interpretability and predictive strength of the findings. Accordingly, the results should be understood as exploratory and hypothesis-generating rather than predictive or causal. Future research should prioritize integrating more complete and higher-quality routine data, including longitudinal adherence indicators, HIV-related clinical variables, socio-economic measures, and more direct indicators of community engagement and clinical governance. In addition, longitudinal, multilevel, and mixed-methods approaches may be better suited to capturing the complexity of DR-TB program implementation and treatment outcomes in high-burden rural settings. Overall, this study contributes useful public health insight by demonstrating both the potential and the limitations of using routine program data to examine DR-TB outcomes. It further underscores the importance of strengthening routine health information systems and governance-sensitive program evaluation approaches to better understand and improve DR-TB care in resource-constrained settings.

## Figures and Tables

**Table 1:** Baseline characteristics.

Characteristic	Value
Age (mean ± SD)	40.69 ± 17.38
Male, *n* (%)	409 (58.9%)
Female, *n* (%)	285 (41.1%)
Treatment success, *n* (%)	416 (59.9%)
Short regimen, *n* (%)	616 (88.8%)
Long regimen, *n* (%)	47 (6.8%)
Severe resistance (Pre-XDR/XDR), *n* (%)	12 (1.7%)

Note: Short and long regimens refer to standardized drug-resistant tuberculosis (DR-TB) treatment regimens implemented within the national tuberculosis program in accordance with World Health Organization (WHO) guidelines.

**Table 2:** Adjusted multivariable logistic regression model for treatment success.

Predictor	Adjusted OR	95% CI	*p*-Value
Constant	1.293	0.395–4.236	0.671
Age (centered)	0.999	0.983–1.015	0.871
Gender	1.334	0.777–2.291	0.296
CE–CG period	0.443	0.240–0.818	0.009
Regimen at initiation	1.220	0.515–2.890	0.652
Severe resistance (Pre-XDR/XDR)	0.303	0.089–1.029	0.056
Any comorbidity	0.935	0.542–1.612	0.808

**Table 3:** Model Diagnostics and Goodness-of-Fit.

Metric	Result	Interpretation
Pseudo-R^2^	0.029	Modest explanatory strength
LR χ^2^	10.70 (*p* = 0.098)	Borderline overall model improvement
VIF	~1.0	No multicollinearity
AUC (previous)	0.52–0.55	Weak discrimination

## Data Availability

The data from this study is available upon request from the corresponding author.

## Abbreviation

DR-TB	Drug-Resistant Tuberculosis
MDR-TB	Multidrug-Resistant Tuberculosis
RR-TB	Rifampicin-Resistant Tuberculosis
XDR-TB	Extensively Drug-Resistant Tuberculosis
Pre-XDR	Pre-Extensively Drug-Resistant Tuberculosis
CE–CG	Community Engagement–Clinical Governance
TB	Tuberculosis
HIV	Human Immunodeficiency Virus
CHW	Community Health Worker
aOR	Adjusted Odds Ratio
CI	Confidence Interval
ROC	Receiver Operating Characteristic
AUC	Area Under the Curve
VIF	Variance Inflation Factor
SD	Standard Deviation
LR	Likelihood Ratio
CE	Community Engagement
CG	Clinical Governance
WHO	World Health Organization
